# Twiddler syndrome mimicking an abdominal aortic aneurysm

**DOI:** 10.1007/s12471-015-0759-5

**Published:** 2015-10-08

**Authors:** K.P. Wevers, L. Kleijn, A.E. Borger van der Burg, M.G. van Andringa de Kempenaer

**Affiliations:** Medical Centre Leeuwarden, Henri Dunantweg 2, PO Box 888, 8901 BR Leeuwarden, The Netherlands

A 79-year-old female was admitted to the emergency department because of a suspected symptomatic abdominal aortic aneurysm. Her medical history reported coronary artery bypass grafting and a DDD pacemaker because of a total atrioventricular (AV) block. Several years later, an upgrade to a biventricular ICD was performed, because of deterioration of the left ventricular (LV) function with symptomatic heart failure, most likely due to 100 % right ventricular (RV) pacing. The RV pacing lead was disconnected.

She presented in a haemodynamically stable condition with a visible pulsating abdominal mass (supplemental video) covering both the central and upper right region of the abdomen without abdominal pain or gastrointestinal complaints. The peripheral pulsations were symmetrically intact.

A computed tomography scan ruled out an abdominal aortic aneurysm. The subsequent chest X-ray showed a mirrored position of the pacemaker and dislocation of the LV pacing lead (Fig. 1a, b) to the superior caval vein, which is anatomically near the right phrenic nerve (Fig. 1b, c). ICD interrogation showed increased lead impedance of the LV lead and failure to capture the left ventricle. After LV lead repositioning the abdominal mass disappeared and patient was discharged.

**Fig. 1:**
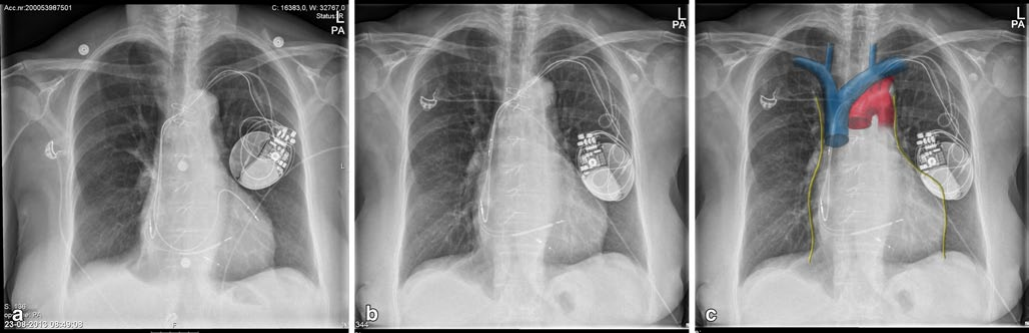
**a** Post implantation chest X-ray. **b** Chest X-ray on admission, mirrored position ICD and dislocated LV lead in vena cava. **c** Chest X-ray on admission with anatomical drawing overlay (phrenic nerve in *yellow*, vena cava superior in *blue*, aorta in *red*) (Anatomical overlay Fig. 1C created by M.R. Wevers).

In conclusion, phrenic nerve stimulation due to LV lead dislodgement can mimic an abdominal aortic aneurysm. Pacemaker rotation leading to lead displacement was previously described by Bayliss et al. as Twiddler’s syndrome [1] and has a reported frequency of 0.07–7 % [2]. It was named after patients who twiddled with the device resulting in rotation and lead dislodgement. However, the syndrome can also occur spontaneously. It causes failure to pace and can result in stimulation of the brachial plexus, vagus nerve, pectoral muscle, or the phrenic nerve [2]. Twiddler’s syndrome is more frequent in older female and obese patients probably due to more subcutaneous space between the cutis and pectoral muscle [3, 4]. Fixating the device at the pectoral fascia can prevent this complication.

## Funding

None.

## Conflict of interest

None declared.

## Electronic supplementary material

(MOV 2160 kb)
